# A Review About the Assessment of the Bleeding and Thrombosis Risk for Patients With Myeloproliferative Neoplasms Scheduled for Surgery

**DOI:** 10.7759/cureus.56008

**Published:** 2024-03-12

**Authors:** Mihaela Andreescu, Bogdan Andreescu

**Affiliations:** 1 Faculty of Medicine, Titu Maiorescu University, Bucharest, ROU; 2 Hematology, Colentina Clinical Hospital, Bucharest, ROU; 3 Plastic Surgery, Colentina Clinical Hospital, Bucharest, ROU

**Keywords:** surgery, antiplatelet therapy, perioperative management, thrombosis risk, myeloproliferative neoplasms

## Abstract

Myeloproliferative neoplasms (MPNs) present a unique challenge in surgical management due to their inherent predisposition to both bleeding and thrombosis. MPNs are a heterogenous group of acquired clonal conditions. The three classic MPNs are essential thrombocythemia (ET), myelofibrosis (PMF), and polycythemia vera (PV). All subtypes of MPN are associated with both thrombotic and bleeding complications. There are four risk categories for thrombosis in MPN patients: age, thrombosis history, and *JAK*-2 mutation. They are further classified as very low, low, intermediate, and high risk. The genetic landscape of MPN is fascinating and complex like all myeloid disorders. Bleeding risk can be assessed through leukocytosis, thrombocytosis, acquired von Willebrand syndrome (AVWS), and a previous history of bleeding in a patient. Risk assessment and perioperative management are important aspects of improving the quality of life and preventing complications in surgeries. Preoperative management includes a risk assessment of venous thromboembolism, use of appropriate pharmacological treatment, platelet count control, and correction and cardiovascular risk factors. This review summarizes the assessment of bleeding and thrombosis risk for patients with MPNs scheduled for surgery. Furthermore, this review discusses various tools that can be used to identify MPN patients at risk of thrombosis prior to surgery.

## Introduction and background

Myeloproliferative neoplasms (MPNs) are a heterogeneous group of acquired clonal disorders. These disorders are characterized by the alteration of myeloid progenitors due to anomalous hematopoietic stem cells, leading to the overproduction of one or more types of myeloid cells. The three primary MPNs include essential thrombocythemia (ET), marked by an overproduction of platelets; myelofibrosis (PMF), characterized by an excess of megakaryocytes leading to increased bone marrow fibrosis; and polycythemia vera (PV), involving an abundance of erythroid cells [1]. Despite their differences, all MPNs share clinical features, such as panmyelosis, splenomegaly, constitutional symptoms, and a heightened risk of bleeding and thrombosis [2]. Thrombosis, venous or arterial, is a major cause of mortality and morbidity in ET and PV, while bleeding is more concerning in PMF and ET [3]. Arterial thrombosis occurs roughly three times more frequently than venous thrombosis, with estimates suggesting rates of 0.7-1.3 per 100 patients per year in PV and 0.5-1.2 per 100 patients per year in ET. MPN patients have a higher rate of recurring thrombosis after the venous or arterial events, which is assessed to be about 6-7.6 per 100 patients per year [4]. A systemic review and meta-analysis reported the Philadelphia-negative MPN diagnostic prevalence at 20% (95% confidence Interval (Cl), 16.6-23.8%; I^2^ 95%); arterial thrombosis at 16.2%, and venous thrombosis at 6.2% [5]. 

MPN patients are also at high risk of developing thrombosis at some unusual sites, such as splenic, portal, and hepatic veins and mesenteric and sinus arteries. In the case of PV, the incidence of thrombotic events can be related to the degree of hematocrit elevation [6]. Recent research has highlighted mechanisms underlying thrombosis in MPNs, including interactions between red cells and platelets leading to increased platelet activation and coagulation factor complex assembly. It was presented that red cell/platelet interactions can lead to increased platelet Fas ligand exposure. This exposure activates death receptors present in the red cells. These receptor/ligand interactions consequence in further externalization of red cells phosphatidylserine. This promotes the assembly of coagulation factor complexes leading to the formation of occlusive thrombi and thrombin generations [7]. Similarly, a study by Guy et al. reported that JAK2V617F MPNs cause thrombosis through the induction of endothelial P-selectin expression. They further revealed that it can be reversed by hydroxyurea, an anti-metabolite that is also used to treat sickle cell disease to reduce vaso-occlusive crises [8].

To avoid thrombotic complications, antiplatelet therapy and cytoreduction therapy are also used in patients with ET and PV. In several patients, disease and genomic-related factors have also been identified that interfere with thrombotic risks. To date, there are no routine laboratory investigations that can accurately assess the core procoagulant state and predict the risks of thrombosis. Traditional coagulation tests, which measure how quickly blood clots, are not always accurate in this regard. However, fibrin generation, thrombin, and thromboelastography (coagulation assays) can provide a more thorough assessment [9]. Whole blood platelet aggression (WBPA) studies are also useful for MPN patients for the evaluation of thrombosis risk [10].

Surgical procedures also pose a significant risk of bleeding in MPN patients, with a probability of 7.2% during surgery [11]. Assessing bleeding and thrombosis risk in MPN patients scheduled for surgery is crucial to optimize patient outcomes. This knowledge can enable the development of personalized management strategies, including the adjustment of antiplatelet therapy, platelet transfusions, or clotting factor administration. The ultimate goal of the present review is the evaluation of bleeding and thrombosis risk for patients suffering from MPNs.

## Review

Risk factors for thrombosis and bleeding in MPN patients

Age

Age is a significant risk factor for thrombosis in MPN patients. An advanced age (above 60 years) is thought to be related to more thrombotic events [12]. However, Hultcrantz et al. reported that the risk of venous and arterial thrombosis was seen significantly elevated in all age groups and was not restricted to only those who were over the age of 60 years [13]. Similar observations related to a high risk of thrombotic events in young MPN patients have also been reported in some studies [14,15]. In a contemporary cohort of 444 young MPN patients, the possibility of thrombosis was in 1% of patients per year [16]. Male predominance is also seen in some studies [10,17]. Due to the limited number of events, further analysis within the younger age groups and genders should be performed and interpreted with caution.

Thrombosis History

Thrombosis history is another risk element for thrombosis in MPN patients, as evidenced by various studies [9,10,18]. Deep venous thrombosis is often triggered by a combination of risk factors, such as pregnancy, hip fractures, immobility, and inherited thrombophilias. The exact mechanism leading to deep venous thrombosis is not fully understood, but recent research has shed light on the role of platelets, endothelium, leukocytes, and venous flow, as well as the interaction between hemostasis and inflammation [19].

MPNs are also described by a rise in particular cell lines, such as anemia. This rise is specific to MF, erythrocytosis is common to PV, while thrombosis is common among all MPNs. However, attempts to estimate the risk of thrombosis based solely on platelet counts or erythrocyte levels have not been successful, especially when considering different MPN subtypes together. In all, MPN subtypes, especially in ET, marked elevated platelet counts are seen. In PV, high platelet counts are thought to be related to a high risk of thrombosis [20]. However, when these elevated levels were adjusted for laboratory and clinical factors, they did not have a significant link with thrombosis [21]. It has been proposed that raised platelet counts can also be a defensive mechanism against thrombosis [21]. These findings suggest that setting a specific target platelet count to reduce thromboembolic risk in ET and PV patients may not be straightforward [20].

Interestingly, the degree of thrombocytosis has not been confirmed to be considerably related to the thrombotic dilemma. Experimental data have proved that there is a noteworthy positive connection between thrombosis and raised white blood cells in both ET and PV [10]. A meta-analysis examined 30,000 patients with ET and PV. The results of this meta-analysis confirmed a 60% elevated risk of thrombosis in the case of leukocytosis. This risk was primarily considered by ET with a comparative risk (risk ratio (RR) of 1.65% Cl, 1.43-1.9), and this RR was only remarkable for arterial incidents (RR, 1.45; 95% Cl, 1.13-1.86) [22]. However, a recent publication that investigated consistently raised white cell counts in PV patients over time was unsuccessful in showing a sequential association between thrombosis and leukocyte trajectory [23]. Hence, the independent influence of leukocytosis as a clinical variable leading to thrombosis in MPN patients remains unclear [20]. In addition, the relationship between blood count elevation in MPNs and thrombosis risk is complicated, particularly in splanchnic vein thrombosis, where factors like portal hypertension and plasma volume expansion can confound the association between blood counts and thrombosis risk [20].

Genetic Mutations

The genetic landscape of MPNs is intricate and constantly evolving. Mutations play a crucial role in both diagnosing and predicting the clinical phenotype of MPNs. Understanding its role in the pathophysiology of the disease is an ongoing process. Mutations, such as JAK2 V617F, play a significant role in diagnosing MPNs and also contribute to prognosis and clinical phenotype [24]. For example, in ET, the presence of JAK2 V617F mutation is associated with up to a two-fold increase in the risk of thrombosis, including both venous and arterial types [20]. This mutation has been extensively studied as a risk factor for thrombosis in various MPN cohorts. In MF and ET, a high risk of thrombotic incidents was observed in patients with JAK2V617F+ compared to those with JAK2V617F- MF or ET. The status of JAK2V617F is now integrated with the international predictive score for thrombosis in ET [25].

For the exploration of risk factors of thrombosis in JAK2V617F-mutated MPNs, a cohort composed of 1,537 Chinese people was retrospectively analyzed. The multivariate analyses found thrombotic events in 675 people, among which 617 were suffering from arterial thrombosis and 112 were suffering from venous thrombosis. The thrombotic events found in PMF, ET, and PV patients were 39, 197, and 439, respectively. The study reported that the JAK2V617F allele burden (V617F%) equal to or greater than 50% is a risk factor for thrombosis in JAK2V617F-mutated MPNs [26]. In addition to its deep-rooted role in thrombosis, the mutations in JAK2V617F are particularly associated with the risk of cardiovascular diseases [27]. Advancements in molecular testing have made it the standard of care for risk stratification and diagnosis of MPNs. Various molecular testing approaches are available, each with different sensitivities for detecting MPN-related mutations [28].

Acquired Von Willebrand Syndrome (AVWS)

AVWS is an enormously heterogeneous group of bleeding disorders characterized by mild to moderate hemorrhagic symptoms. While the severity of symptoms can vary, they can be significant, particularly during surgical procedures. AVWS is not inherited like inborn von Willebrand disease but rather occurs sporadically, often associated with conditions, such as myeloproliferative, cardiovascular, and lymphoproliferative disorders [29-31].

In a recent study, Song et al. analyzed the clinical features of AVWS in all subtypes of MPN. AVWS was found in 31.3% of patients and was more frequently present in ET patients (41.4%). The percentage of AVWS was 33.3% and 17.6% in pre-PMF and PV patients, respectively. The relation between AVWS and bleeding was not fully understood as only minor episodes of bleeding were seen in one ET patient with AVWS [32]. This highlights the unique hemostatic imbalance observed in ET, where both thrombosis and bleeding can occur simultaneously [31].

Similar results were found in another study that was performed to screen the AVWS in patients with myelofibrosis and its association with bleeding risk. Only a single major bleeding event was reported in a cohort of 60 patients [33]. In a recent report, a young man without any genetic mutation and with ET had paradoxical bleeding due to AVWS. This paradoxical bleeding was the result of AVWS being an entirely distinct entity from the hyperthrombic state [34]. An additional study of low-risk ET defined by the WHO did not find a major difference in bleeding rates between controlled and aspirin-treated patients (P = 0.2). In the case of mutation status, there was a similar rate of bleeding without and with aspirin in patients with JAK2-positive patients. Calreticulin (CALR)-mutated patients had shown a higher risk of bleeding with aspirin than in the observational group (P = 0.03) [35]. Hence, careful assessment of laboratory markers and clinical symptoms is required to diagnose AVWS as a complication and risk assessment factor for bleeding.

Leukocytosis

Leukocytosis can also be a risk factor that should be assessed for bleeding in MPN patients. A previous study conducted for ET and PMF found that leukocytosis is an independent risk factor for bleeding (P = 0.041) with a 2.0% patients per year bleeding rate in PMF [36]. Another study of ET found a link between the risk of bleeding and leukocytosis. An exploration of ET patients introduced a U-shaped association between the risk of hemorrhage and white blood cells. The elevated leukocytes have a high risk of bleeding [37]. There is another study that contradicts these results by reporting no link between leukocytosis and the risk of bleeding [35]. Hence, further investigations are required to confirm leukocytosis as a risk for bleeding in MPN patients.

Thrombocytosis

Thrombocytosis is one of the risk elements for bleeding in the MPN patients. This has been proved by a longitudinal cohort study, whose results on ET reported that an increased risk of bleeding is associated with abnormally high platelet counts as compared to the normal platelet count range. The hazard ratio found in this study was (HR 3.7%; 95% CI 1.7-8.2) [37]. The role of abnormal platelet count and surface receptors is still a topic of debate. There is evidence that JAK2V617F has a direct effect on the platelet stimulation in ET through a very intricate mechanism of the phosphoinositide 2-kinase (PI2K)/Ras-proximate-1 (Rap1) path priming to the compromised thrombopoietin-mediated integrin IIB3 activation. Moreover, in MF patients, thrombocytopenia and hypersplenism may also have a high risk of bleeding [38].

Despite several studies conducted in the past, there is a lack of best evidence to withstand the currently available evidence of an association between increased risk of bleeding and thrombocytopenia. Surgeries can be conducted safely when the thrombocyte level is not too high. Therefore, adequate perioperative care is important for the prognosis of ET patients undergoing surgery [39]. Preoperative management includes a risk assessment of venous thromboembolism, the use of appropriate pharmacological treatment, platelet count control, and correction and cardiovascular risk factors [39]. Elective surgery should be delayed until platelet counts return to normal levels and any cardiovascular risk factors are addressed. Symptomatic ET patients may require cytoreductive therapy to achieve this, while perioperative thromboprophylaxis may be necessary in emergency cases [40].

Risk assessment tools

Rational Elastrometry (ROTEM)

ROTEM has evolved from the thromboelastometry technology (TEG). Both TEG and ROTEM are viscoelastic tests of hemostasis that allow measurements of global clot formation and dissolution time in real time. ROTEM provides a visual evaluation of clot formation and succeeding lysis under low-shear conditions similar to those present in the vena cava, venules, arterial system, and large veins [41]. ROTEM has proven valuable for diagnosing thrombotic and bleeding disorders, including in patients with MPNs. It provides rapid and complete information about all the stages of the coagulation process. A recent study has shown that ROTEM can identify MPN patients at risk of hemorrhagic or thrombotic complications [42]. Another study showed that ROTEM analysis of MPN patients presented a hypercoagulation profile who had a higher maximum clot firmness and shorter clot formation time as compared to the control group. ROTEM displays a hypercoagulative state in PV and ET patients, and its parameters are significantly influenced by platelet counts [43]. However, further research is needed to establish ROTEM's predictive value for identifying high-risk patients.

International Predictive Score for Thrombosis in ET (IPSET)

IPSET is a risk assessment tool specifically designed for ET patients. It categorizes patients into four risk groups based on three factors: age, presence of JAK-2 mutations, and history of thrombosis. These risk groups are labeled as low, very low, intermediate, and high risk [44]. Validation tests have been performed by various studies to confirm the effectiveness of this model. In a previous study, the revised IPSET-Thrombosis was validated through 585 ET patients. This model provided a significant difference in risk of thrombosis between low and very low (HR 2.4, 95% CI 1.1-5.3) and between intermediate and high-risk patients (HR 2.3, 95% CI 1.1- 5.3). However, the revised IPSET-thrombosis needs confirmation through prospective studies, especially in the case of risk-adapted therapy [45]. IPSET scores are convenient for being used in the risk stratification of thrombosis in pre-PMF patients. This may symbolize the base of personalized management focused on decreasing the elevated risk of chief cardiovascular incidents. However, additional improvement of the IPSET score was followed by further potential studies assessing the presence of adversative mutational profiles as unusual variables or leukocytes [46].

DIPSS-Plus

The dynamic international prognostic scoring system (DIPSS) places patients in one out of four risk categories. These categories include high, intermediate-1, intermediate-2, and low risks. This prognosis is determined by red blood cell transfusions, thrombocytopenia, and karyotypes. The addition of these three factors composes DIPPS into DIPSS plus. This predictive tool was developed originally for MF patients only. This tool is also being applied for scoring in patients with secondary MF [47].

Surgical consideration in MPN patients

MPNs can complicate the surgical process due to spontaneous bleeding and thrombosis. However, currently, there are no concrete guidelines or prerequisites for the patients of ET who undergo surgery [39]. It has been reported that ET patients have a higher risk of arteriovenous thrombosis and risk of bleeding by 500% and 10.5%, respectively [48]. After analyzing a case report of 48-year-old women, it was concluded that surgery in MPN patients with elevated thrombocytes can be considered only if the benefits outweigh the adverse risks [39]. A recent study by Gurrieri et al. suggested that a thrombocyte count above 800,000/mm^3^ has an association with an increased risk of thrombotic complications. Surgeries are considered to be safe when patients have a thrombocyte count below 450.000/mm^3^. On the other hand, another study reported that surgeries were conducted in ET patients without any hemostasis complications despite high thrombocyte counts of 280,000/mm^3^ and 313.000/mm^3^ [49,50]. Hence, a low thrombocyte count did not assure the development of hemostasis complications, and adequate perioperative care is important for the prognosis of ET patients who underwent surgeries [49].

Management of thrombosis and bleeding in MPN patients

Management of Thrombosis

There are different strategies for the management of thrombosis in all three types of MPN on the basis of risk levels. Thrombosis management is described briefly in Table 1.

**Table 1:** Thrombosis management based on risk levels in MPN patients PV: polycythemia vera, ET: essential thrombocythemia, PMF: primary myelofibrosis, IFN: interferon

Management of PV		
For all patients	Low risk	High risk
One aspirin per day [51]; phlebotomy to keep hematocrit less than 45% [52]	Two aspirin per day if microvascular symptoms are not under control and cardiovascular risk factors and leukocytosis occurs [53]	Cytoreduction with hydroxyurea; consider second-line drugs if hydroxyurea is intolerant or resistant (busulfan and ruxolitinib) [54]
Management of ET		
Low risk	Intermediate risk	High risk
If no cardiovascular risk is found, one aspirin per day. If found, consider two aspirin per day [53].	One aspirin per day if no cardiovascular risk factors are found. For cardiovascular risk factors, consider cytoreduction with hydroxyurea and one aspirin per day [55].	Add anticoagulants for venous thrombosis history; consider two aspirin per day for arterial thrombosis; consider second-line drugs if hydroxyurea is intolerant or resistant (busulfan, ruxolitinib, and INF-α) [56].
Management of PMF		
Low risk	Intermediate risk	High risk
Observation and one aspirin per day [57]	One aspirin per day for arterial thrombosis; anticoagulants for leukocytosis and venous thrombosis; avoid using cytoreductive therapy, including the use of hydroxyurea or ruxolitinib, for MF-associated anemia [58]	Cytoreduction with hydroxyurea for leukocytosis and thrombocytosis [58]

Management of Bleeding

For the effective management of bleeding in MPN patients, several factors should be considered some of these factors, and their treatment options are listed in Table 2.

**Table 2:** Management of bleeding in MPN patients in certain conditions AvWS: acquired von Willebrand syndrome

Conditions	Management
Management of bleeding risk in the presence of VWS activity <30% and aVWS	Avoid aspirin, antiplatelet therapy [31]
Extreme thrombocytosis	Avoid aspirin, cytoreductive therapy [59]
Other medical management of bleeding includes platelet transfusions, antifibrinolytic agents, factor replacement, and desmopressin	

## Conclusions

All subtypes of MPN are associated with both thrombotic and bleeding complications. The management of these complications depends on risk stratifications centered on identifiable risk factors. The identifiable risk factors for thrombosis are age >60 years, a previous thrombosis history, and genetic mutations, particularly variants of JAK2V617F. These risk factors are identified by some studies performed in the past, while some showed no association of these factors toward thrombosis in MPN patients. Thrombosis risks can be assessed through IPSET. The same is true for the risk of bleeding. The identifiable risk factors for bleeding were leukocytosis, thrombocytosis, AVWF, and a previous history of bleeding. Due to a high risk of bleeding and thrombosis in MPN patients, reassessment of antiplatelet, cytoreductive, and anticoagulant treatments is required. Individual patient factors must be considered to minimize severe bleeding and thrombotic complications in surgeries. Risk assessment and perioperative management are important aspects of improving the quality of life and preventing complications in surgeries. Further analysis and studies are required for the confirmation of risk assessment factors for both thrombosis and bleeding events in MPN patients.
